# Combined Hormonal Contraception during Breastfeeding—A Survey of Physician’s Recommendations

**DOI:** 10.3390/jcm12227110

**Published:** 2023-11-15

**Authors:** Lior Segev, Gideon Weitzman, Goldie Katz-Samson, Abraham O. Samson, Guy Shrem, Naama Srebnik

**Affiliations:** 1 Azrieli Faculty of Medicine, Bar Ilan University, Safed 1311502, Israel; shgev@puah.org.il; 2 PUAH Institute: Fertility, Medicine, Halacha, Jerusalem 9547735, Israel; 3 Nishmat: The Jeanie Schottenstein Center for Advanced Torah Study for Women, Jerusalem 9328249, Israel; 4 IVF Unit, Department of Obstetrics and Gynecology, Kaplan Medical Center, Rehovot 7610001, Israel; dr.shremguy@gmail.com; 5 Faculty of Medicine, Hebrew University of Jerusalem, Jerusalem 9112102, Israel; naama.srebnik@mail.huji.ac.il; 6 IVF Unit, Department of Obstetrics and Gynecology, Shaare Zedek Medical Center, Jerusalem 9103102, Israel

**Keywords:** combined hormonal contraception, progesterone-only pill, breastfeeding

## Abstract

Until breastfeeding is established, progesterone-only pill (POP) use is preferable over combined hormonal contraception (CHC), as the latter potentially reduces milk production. Yet, POPs are often associated with breakthrough bleeding (BTB), and irregular spotting is often a reason for their cessation. Conversely, CHC is less associated with BTB but is not usually prescribed, even if breastfeeding has been established, despite its verified safety profile. Here, we surveyed physicians’ perception of CHC safety during breastfeeding through an online questionnaire (N = 112). Physicians were asked if they would prescribe CHC to a woman three months postpartum, breastfeeding fully, and suffering from BTB while using POPs. Half of the physicians responded they would, 28% would not until six months postpartum, while 14% would not during breastfeeding. Of the physicians that would prescribe CHC, 58% would without any reservation, 24% would only after discussing milk reduction with the patient, 9% would use a pill with a lower hormonal dose, and 9% would only prescribe CHC 3 months postpartum. The main risk associated with CHC during breastfeeding, as perceived by physicians, is a potential decrease in breast milk production (88%). While some physicians consider CHC unsafe during breastfeeding, most health organizations consider CHC compatible with breastfeeding 5–6 weeks after birth. Thus, there is a gap in the attitude and knowledge of physicians about the safety profile of CHC, and only half acknowledge that the risk of BTB justifies the use of CHC instead of POPs while breastfeeding three months postpartum. We highlight the importance of physician’s education, advocate CHC breastfeeding compatibility if breastfeeding has been established (i.e., 30 days postpartum), and underline the importance of discussing the option of CHC with patients in case POPs have unwanted side effects.

## 1. Introduction

In the period immediately following childbirth, the establishment of successful breastfeeding holds paramount importance. Until breastfeeding is established, it is recommended that progesterone-only pills (POPs) be favored over combined hormonal contraception (CHC) as the latter has the potential to impede milk production, as pointed out in various medical studies [1]. Nevertheless, the use of POPs comes with its own set of challenges. This method has been associated with a notably higher incidence of breakthrough bleeding (BTB) and irregular spotting [2], which, though not posing any direct medical risks, can considerably disrupt a woman’s daily life. Despite its seemingly benign nature, the impact of BTB should not be underestimated. Many women grapple with significant discomfort and inconvenience due to BTB, leading to approximately 25% discontinuing the use of POPs altogether [3], Consequently, this discontinuation significantly heightens the risk of conception occurring shortly after giving birth, potentially impacting the woman’s health and the well-being of the newborn.

Moreover, for certain religious communities, BTB holds a profound cultural and religious significance. Among Orthodox Jews, this bleeding has a religious bearing, as Jewish law prohibits any physical intimacy up to 12 days after the last bleeding [4]. Consequently, the occurrence of BTB within this community can have significant implications on the daily life and intimate relationships of individuals adhering to this religious practice. Similarly, among devout Muslims, Islamic law prohibits sexual intercourse during periods of bleeding, further highlighting the importance of managing BTB among this religious group [5]. Thus, the management of BTB becomes not only a medical concern but also a culturally and religiously significant issue within these communities.

Conversely, combined hormonal contraception (CHC) is less associated with breakthrough bleeding (BTB) compared to progesterone-only pills (POPs). Despite this advantage, CHC is not often prescribed even if breastfeeding is established, even though its safety profile has been well established. Research has indicated that CHC could potentially lead to a reduction in breast milk production, as indicated in some studies [1]. However, the data on the use of CHC during breastfeeding remain conflicting, leaving medical practitioners and patients uncertain about its suitability in these circumstances. The debate surrounding the use of CHC during breastfeeding is characterized by conflicting reports and a lack of consensus. Thus, while these potential concerns are legitimate and until breastfeeding is established, progesterone-only pills (POPs) are often preferred over combined hormonal contraceptives (CHCs) despite their established safety profiles [6].

Here, we assess the attitude and practice of physicians regarding the use of CHC during breastfeeding, particularly amongst women for whom BTB is a serious non-medical concern while using POPs. This study expands on our earlier findings published in breastfeeding medicine [7].

## 2. Materials and Methods

An online questionnaire was sent to physicians through social media private groups for physicians (WhatsApp), including approximately 300 participants. There were no inclusion or exclusion criteria for selecting the participating physicians in this study. The questionnaire was shared in groups of general practitioners (GPs) and gynecologists (referred to as OB-GYNs—obstetrics and gynecology) practicing throughout Israel. The cohort size was unlimited and included all practitioners who answered without any limiting factors. We also included general physicians in an attempt to compare practices in different medical disciplines. Since there were no inclusion criteria, the group is expected to include a random cohort with no conflicts of interest.

The study received approval from the Ethics Committee of the Faculty of Medicine of Bar Ilan University (IRB 01-2023).

The questionnaire included demographic questions: gender, specialty (GP or OB-GYN), years of experience (<10 years, 10–20 years, or >20 years), and occupational location (central cities/periphery). Then, the questionnaire presented a case of a woman three months postpartum who suffers from BTB while using POPs and requests CHC. The questionnaire offered four choices: (1) The physician agrees to prescribe CHC. (2) The physician does not agree to prescribe CHC. (3) The physician agrees to prescribe CHC, but only six months postpartum. (4) Other. Physicians were asked to state the main risks associated with CHC during breastfeeding and whether they had any reservations about CHC safety (using free text). Next, a short paragraph including data on the safety of CHC during breastfeeding was presented, and physicians were asked whether they would change their recommendation in the case presented and if they wanted to learn more. 

## 3. Results

A total of 112 physicians answered the questionnaire (37% response rate). The demographic data of the physicians is listed in Table 1. Both general practitioners and gynecologists were surveyed to examine whether the discipline affected physicians’ attitudes and knowledge.

**Figure 1:**
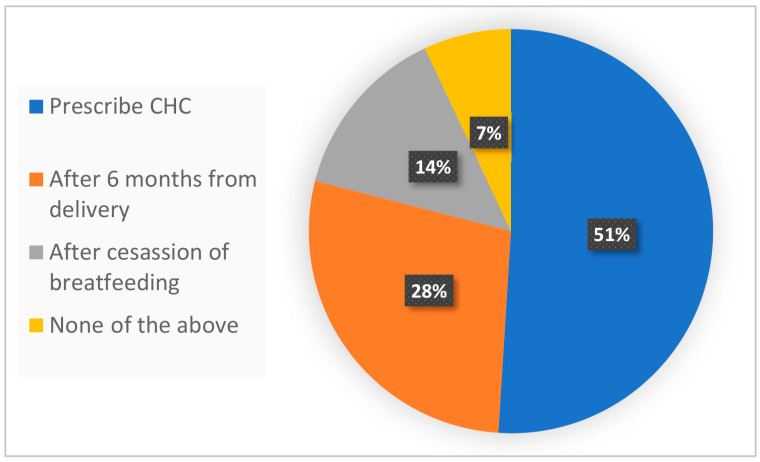
Physician’s responses to the case presented. CHC—combined hormonal contraception.

Figure 1 presents our survey results and shows the perceived CHC compatibility in the case of a patient, three months postpartum and fully breastfeeding, suffering from BTB while using POPs. Remarkably, 51% of the physicians responded they would prescribe CHC, while 28% would not until six months after birth, and 14% would not until after the cessation of breastfeeding. A minority of physicians (7%) responded “other” and likely disagreed with any of the available responses (Figure 1). The results illustrate the discrepant views among physicians of CHC compatibility three months after birth. 

Table 2 presents the survey’s results according to the different demographic characteristics of the physicians. The response variance among different demographics is statistically insignificant (average Pearson correlation coefficient is 0.94, *p*-value > 0.05), and the perceived CHC compatibility during breastfeeding is independent of physician gender, medical specialty, and years of experience.

Table 3 shows the reservations among physicians who would prescribe CHC in the case presented. These reservations are provided by physicians as freestyle text and grouped into four categories, as shown in Table 3. Notably, among prescribing physicians, 58% did not have any reservations, 24% would prescribe CHC only after discussing potential side effects like breast milk reduction, and 9% would prescribe a lower CHC dose. Table 3 also shows the physician’s responses according to their medical specialty. Interestingly, among CHC prescribers, OB-GYNs were more careful than general practitioners. Unlike 75% of the general practitioners, only 55% of the OB-GYNs did not provide any reservations. Unlike none of the general practitioners, 29% of OB-GYNs would discuss potential side effects before prescribing CHC, and 10% would not prescribe CHC before three months after birth. OB-GYNs are also more careful than general practitioners in using the standard therapeutic dose. Unlike 25% of general practitioners, only 6% of OB-GYNs would prescribe a low and potentially non-therapeutic dose of CHC. Thus, 58% of prescribing physicians did not cite any reservations. 

**Figure 2:**
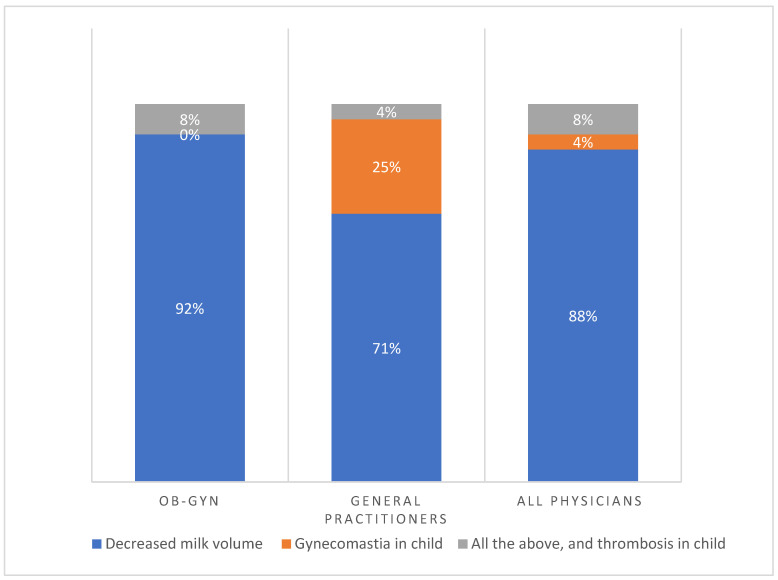
The main risks of CHC during breastfeeding, as perceived by physicians according to medical specialty. CHC—combined hormonal contraception, OB-GYN—obstetrics and gynecology.

**Figure 3:**
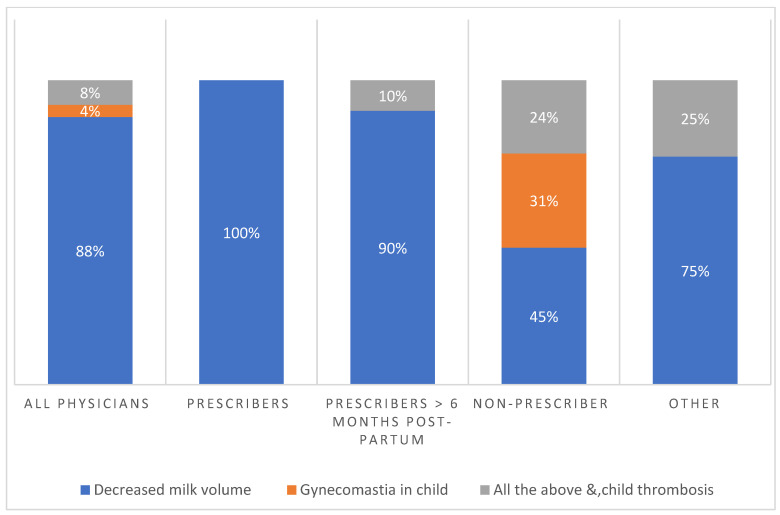
According to the preliminary answers, the main risk of combined hormonal contraception during breastfeeding as perceived by physicians.

Figure 2 shows the main risks of CHC during breastfeeding as perceived by physicians according to medical specialty. Notably, the main reason provided was decreased breast milk volume (88%). Less common reasons provided were the risk of gynecomastia in the nursing infant (4%), a combination of risks, and thrombosis in the nursing child (8%). Interestingly, 92% of OB-GYNs were primarily concerned about the risk of milk reduction compared to 71% of general practitioners. Unlike 25% of general practitioners, no OB-GYNs perceived gynecomastia in children as the main risk and reason for avoiding CHC during breastfeeding. Some 8% of OB-GYNs were also concerned about a combination of all risks compared to 4% of general practitioners. Thus, among all physicians, the principal reason for avoiding CHC was the risk of breast milk reduction. Figure 3 shows, according to the preliminary answers, the main risk of combined hormonal contraception during breastfeeding as perceived by physicians. Most prescribers feared decreased milk volume with the use of CHC. 

## 4. Discussion

The attitudes, knowledge, and practice of physicians regarding CHC general use, though not during breastfeeding, were surveyed in several studies. Gaps in knowledge about the limits and contraindications of CHC [8], as well as in the attitude and practice of gynecologists, affecting their preferred method of CHC were found [9]. In line with this context, this current study assessed the attitudes, knowledge, and practice of physicians involved in the care of women postpartum when choosing methods of contraception. Notably, the study uncovered a distinct divide among physicians regarding the prescription of CHC during breastfeeding. While 51% of the physicians surveyed were open to the use of CHC during breastfeeding, a significant portion, constituting 42%, refrained from prescribing CHC even when the woman experienced breakthrough bleeding (BTB) associated with progesterone-only pills (POPs). Of the 42% that would not prescribe CHC, a subset of physicians (28%) indicated a preference for delaying CHC prescription until six months postpartum, while a minority (14%) refused to prescribe CHC for the entire duration of breastfeeding. An intriguing observation was the lack of a discernible correlation between these varied perspectives and the medical specialties, gender, or years of experience of the physicians surveyed. This discrepancy in viewpoints among physicians may be attributed to the conflicting recommendations outlined in different clinical guidelines, which could lead to uncertainty and divergence in clinical practice. The findings of this study shed light on the complexities inherent in the decision-making process of physicians when it comes to prescribing CHC, especially in the context of breastfeeding. Addressing the existing discrepancies and promoting a better understanding of the factors influencing the choice of contraceptive methods among physicians could pave the way for more unified and evidence-based practices in this critical area of women’s health.

Despite a relatively large number of studies dedicated to this topic, a Cochrane review of 11 studies examining various forms of contraception during breastfeeding concluded that there is no definitive answer regarding the potential impact of CHC on breast milk volume [10]. This highlights the need for further research and a more nuanced understanding of the interactions between CHC and breastfeeding.

As an example of conflicting reports, two of the reviewed studies which used similar randomized control trials (RCT) for comparing POPs with CHC reached opposite conclusions, further adding to the complexity of the matter [1,11].

In a noteworthy study conducted by Tankeyoon et al., a comparison was drawn between 86 breastfeeding participants receiving combined hormonal contraception (CHC), consisting of 30 mcg Ethinyl Estradiol (EE) and 150 mcg Levonorgestrel, and 85 participants receiving progesterone-only pills (POPs) with 75 mcg Norgestrel. The researchers meticulously assessed milk volume by employing breast pumping techniques while simultaneously monitoring the growth of the infants involved in the study. The results of the study underscored a trend toward decreased milk production among the participants using CHC. Within six weeks from the commencement of CHC use, there was a noticeable reduction in milk volume among this group. In stark contrast, no such reduction was observed within the group administered with POPs. After 18 weeks, the disparity became even more pronounced, with the CHC group experiencing a substantial 42% reduction in milk volume, whereas the POP group experienced a comparatively moderate reduction of only 12%. Remarkably, the control group, comprising individuals who did not utilize any form of hormonal contraception, demonstrated a minimal decrease of 6% in milk volume. The study concluded that CHC use resulted in a significant decrease in milk production. Despite these significant findings, the study did not uncover any adverse effects on infant growth among the groups under investigation. The researchers carefully evaluated multiple indicators, including body weight, height, arm circumference, triceps skinfold thickness, and head circumference, and noted no substantial variations between the groups. Acknowledging the potential for compensatory measures, the authors suggested that the women may have offset the reduction in milk production by implementing supplementary feeding or by prolonging and intensifying suckling episodes. However, it is essential to recognize that a potential limitation of this study lies in the absence of self-reported data on the frequency of breastfeeding, which could have provided valuable insights into the compensatory mechanisms employed by the participants [1].

In contrast, another study conducted by Espey et al. compared the effects of combined hormonal contraception (CHC) and progesterone-only pills (POPs) on breastfeeding among 64 participants who received CHC containing 35 mcg Ethinyl Estradiol (EE) and 1 mg Norethindrone, and 63 participants who were administered POPs with 350 mcg Norethindrone. The study monitored the participants for a duration of eight weeks while assessing the impact of the contraceptive methods on breast milk production. Surprisingly, the results of this study exhibited a strikingly similar reduction in milk volume within both the CHC and POP groups, with reductions of 63% and 64%, respectively. This outcome contradicts the prevailing notion that CHC may have a more pronounced effect on breastfeeding compared to POPs. Similar to the findings in the previous study, no discernible differences were observed in the growth of the children, as measured by indicators such as infant weight, height, and head circumference. Furthermore, the choice between CHC and POPs did not seem to have any detrimental impact on the continuation of breastfeeding among the participants [11]. As a potential limitation, this study suffers from a subjectivity bias due to the self-reported breast milk production by participants. Despite this potential constraint, the study’s findings provide valuable insights into the comparable effects of CHC and POPs on breastfeeding, suggesting that both methods may have similar implications for breast milk production.

The disputed CHC effect on milk reduction is also evident from the different published recommendations. On the one hand, the World Health Organization (WHO) stringently categorizes CHC during breastfeeding as moderately safe from 6 weeks to 6 months postpartum and as safer after that (>six months) [6]. Likewise, the Royal College of Obstetricians and Gynaecologists (RCOG) recommends the use of CHC only after six months postpartum [12]. On the other hand, the American College of Obstetricians and Gynecologists (ACOG) recommends using CHC starting at five weeks postpartum [13]. Likewise, the British National Health Service (NHS) allows CHC starting at six weeks postpartum, even while breastfeeding [14]. Finally, the Center for Disease Control and Prevention (CDC) categorizes CHC during breastfeeding as safer starting from 42 days postpartum [15].

Another concern associated with CHC is the potential transfer of estrogens, such as Ethinyl Estradiol (EE), into maternal milk. In one study that tested direct transmission, Nilsson et al. examined the presence of EE in milk after exposing participants to either 50 mcg EE or 500 mcg EE. No trace of EE in the 50 mcg EE group was detected in the milk. In the 500 mcg EE, group trace amounts of EE, less than 100 ng, or 0.02% of the original dose, were detected in the milk [16]. Another study examined the passage of EE to the milk 2 h after administrating 0.1 mg of EE, compared to milk drawn immediately following birth, the colostrum. No significant trace of EE was found in breast milk [17]. In another study, 50–200 mcg of Estradiol (E2) was administered before breastfeeding, and E2 levels were then measured in the infants. The study did not observe any increased E2 level in the infants nor any effect on their growth. The researchers concluded that no significant amount of E2 is transmitted to infants through breastfeeding [18].

A final concern associated with CHC is the potential change in maternal milk composition. One study showed that women using different doses of CHCs (EE and Levonorgestrel) expressed various levels of proteins and nitrogen compounds in their milk, although within the normal range [19]. However, another paper found no change in the content of fat, protein, or lactose in milk despite the use of CHC [20].

Recently, a comprehensive review by Lerner and coworkers published in *Breastfeeding Medicine* provided current and updated guidelines on the safety profile of hormonal contraception during breastfeeding. The review concluded that combined hormonal contraception is justified if breastfeeding has been established (i.e., 30 days after birth) [21].

To the best of our knowledge, there are no studies dealing with the gap of physicians’ knowledge of the correct use of combined oral contraception during breastfeeding.

The findings presented in our study reflect the contrasting conclusions surrounding the impact of combined hormonal contraception (CHC) on breast milk production, as recently reviewed by Tepper et al. [22]. In their review of 925 papers listed on PubMed, only 13 met the inclusion criteria of significance. These selected studies compared nursing mothers using CHCs with those using nonhormonal or no contraception at all; in addition, the studies investigated the effects of early initiation of CHCs (<6 weeks postpartum) versus late initiation (>six weeks postpartum) following childbirth. As outlined in their review, most studies demonstrated inconsistent effects of CHC on breastfeeding performance. Some studies indicated a decrease in breastfeeding and an increased need for supplementation among CHC users in comparison to nonusers, while others did not establish any significant effect. A subset of studies found decreased infant weight gains, but only if CHC was initiated before six weeks postpartum. For infant outcomes, other studies found no effect. Remarkably, none of the studies found an effect on infant weight gain if CHC was started later than six weeks postpartum, and no studies found any impact on other infant health outcomes regardless of the time of CHC initiation. Consequently, it can be inferred that the initiation of CHC after the six-week postpartum period does not appear to negatively influence milk production, a stance that is widely endorsed by most health organizations worldwide. Notably, the American Centers for Disease Control and Prevention (CDC), the American College of Obstetricians and Gynecologists (ACOG), and the British National Health Service (NHS) have all adopted this perspective [13,14,15].

On the other hand, the World Health Organization (WHO) references the study by Tankeyoon et al., conducted in Hungary and Thailand, which revealed a substantial 41% reduction in breast milk volume among users CHC [1]. It is plausible that the WHO recommendation is tailored toward developing countries where access to sufficient food sources for infants might be limited. In such resource-constrained settings, the use of CHC could potentially exacerbate the challenge of inadequate milk production for up to six months postpartum, ultimately impacting the nutritional well-being of infants [6]. It is important to acknowledge the need for context-specific approaches to address the complex interplay between contraception, maternal health, and infant nutrition in resource-limited settings. Certainly, in situations where lactating mothers experience challenges with milk production, the use of supplements or galactagogues (such as herbal tea) can potentially play a vital role in enhancing and sustaining milk production in lactating mothers [23]. These supplements and herbal remedies are known for their traditional use in promoting lactation and are often considered safe for consumption by breastfeeding mothers.

The benefits of exclusive breastfeeding, as opposed to partial breastfeeding, remain a topic of ongoing investigation, with findings that have led to varying conclusions. On the one hand, a study in India found that people who were partially breastfed in infancy suffered from a higher incidence of obesity throughout their lives [24]. On the other hand, a study carried out in China did not reveal any significant differences between infants breastfeeding exclusively and those breastfeeding partially [25]. These contrasting outcomes underscore the complexity of understanding the precise implications of exclusive versus partial breastfeeding on long-term health outcomes. Given this context, our study could emphasize the perceived importance of exclusive breastfeeding, and the perceived benefits could also prompt physicians to avoid prescribing CHC during breastfeeding. Indeed, the existing medical literature strongly advocates for exclusive breastfeeding for the first six months of a child’s life. However, it is crucial to acknowledge that the practice of exclusive breastfeeding may not necessarily demonstrate an outright advantage in all contexts [26]. This understanding is important in guiding healthcare providers in their decision-making processes, ensuring that they remain informed about the complexities surrounding breastfeeding practices and their associated health implications. Furthermore, this awareness can empower physicians to provide comprehensive and well-informed guidance to new mothers, promoting optimal health outcomes for both the mother and the child.

POPs are associated with a high incidence of breakthrough bleeding (BTB), and there is a concern that women will cease its use [2]. One study found that 35% of women who stop POPs attribute the cause to BTB and irregular bleeding [27]. In addition to the lifestyle disadvantage, BTB prohibits any intimacy in Jewish and Muslim religious couples. Some women will choose to insert an intrauterine device, but this is not always a viable option for women seeking to use contraception for a limited time. Relying on breastfeeding as the only form of contraception is also unreliable [28,29], and such women could cease using POPs and risk becoming pregnant soon after giving birth. As such, we suggest that physicians discuss the advantages and disadvantages of CHC when there is a concern for the reduction in milk production and in light of the benefits of CHC on BTB. In the past, we have proposed the addition of norethisterone to the POP regimen to reduce the side effects of BTB [30]. This treatment may also be discussed as a treatment option for BTB management.

A potential limitation of this study is the relatively low response rate. However, it is possible that not all physicians were exposed to the questionnaire because it was publicized and distributed through social networks. As another potential limitation of this study, these views reflected herein are of local physicians and could be different elsewhere. Importantly, we note that CHC is contraindicated in women predisposed to thrombosis, cardiovascular disease, smoking, rheumatic diseases, migraines with aura, prolonged immobility, active liver disease, active cancer, and advanced age [6].

In conclusion, we show a range of practices among medical professionals regarding CHC use during breastfeeding. Some 51% of the physicians would prescribe CHC to a woman breastfeeding fully, three months postpartum, and complaining about breakthrough bleeding with POPs. Some 28% of the physicians would not before six months postpartum. Finally, 14% would not permit the use of CHC throughout the period of breastfeeding. In light of this questionnaire, 28% of the physicians were interested in learning more, underlining the need to further investigate and disseminate the advantages and disadvantages associated with CHC during breastfeeding so as to assist physicians and patients with choosing the best pregnancy prevention option.

Our study stresses the importance of ongoing medical education for practicing physicians, particularly in the choice of oral contraception during breastfeeding. While CHC may reduce breast milk production, it does not affect infant growth. Thus, CHC is justified if breastfeeding has been established (i.e., 30 days postpartum) [21]. Ongoing education is important, and the lack thereof can lead to shortcomings and disparities in contraception counseling [31]. As such, we encourage more studies that examine potential pitfalls in contraception counseling [32].

## Figures and Tables

**Table 1:** Physician’s demographic characteristics (OB-GYN—obstetrics and gynecology).

N	112
Specialty	
General practitioner	21 (19%)
OB-GYN	91 (81%)
Gender	
Male	36 (32%)
Female	76 (68%)
Practice	
<10 years	39 (35%)
10–20 years	36 (32%)
>20 years	37 (33%)
Area	
Capital City	57 (55%)
Large Cities	25 (22%)
Periphery	25 (23%)

**Table 2:** Survey results divided by demographics.

Question:	A Patient Presents Three Months Postpartum, Fully Breastfeeding. For the Past Six Weeks, She Received POP and Complains about BTB. The Patient Requests to Change to CHC. Would You Prescribe CHC?				
Answer:	Yes	Yes. But Only 6 Months Postpartum	No. Not While Breastfeeding	Other	Total (N)
All physicians	57 (51%)	31 (28%)	16 (14%)	8 (7%)	112
Medical specialty					
GP	8 (38%)	5 (24%)	4 (19%)	4 (19%)	21
OB-GYN	49 (54%)	29 (26%)	12 (13%)	4 (4%)	91
Years of practice					
<10 years	19 (49%)	13 (33%)	4 (10%)	3 (8%)	39
10–20 years	18 (50%)	10 (28%)	6 (17%)	2 (5%)	36
>20 years	20 (54%)	8 (22%)	6 (16%)	3 (8%)	37
Gender					
Male	19 (52%)	7 (19%)	8 (22%)	2 (7%)	36
Female	38 (50%)	24 (32%)	8 (11%)	6 (7%)	76

CHC—combined hormonal contraception, GP—general practitioner, OB-GYN—obstetrics and gynecology.

**Table 3:** Reservations among physicians prescribing CHC.

Question:	Do You Have Any Reservations about CHC Use?				
Answer:	None	Allow Only 20 mcg EE, But Not 30 mcg	Allow Only after Discussing Milk Reduction with the Patient	Allow Only 3 Months after Birth	Total (N)
Prescribing physicians	33 (58%)	5 (9%)	14 (24%)	5 (9%)	57
Medical specialty					
GP	6 (75%)	2 (25%)	-	-	8
OB-GYN	27 (55%)	3 (6%)	14 (29%)	5 (10%)	49

CHC—combined hormonal contraception, EE—Ethinyl Estradiol, GP—general practitioner, OB-GYN—obstetrics and gynecology.

## Data Availability

The data presented in this study are available on request from the corresponding author.

## Abbreviations

BTB—breakthrough bleeding; CHC—combined hormonal contraception; EE—Ethinyl Estradiol; IUD—intrauterine devices; POP—progesterone-only pill; RCT—randomized control trials; WHO—World Health Organization.

## References

[1] Tankeyoon M., Dusitsin N., Chalapati S., Koetsawang S., Saibiang S., Sas M., Gellen J.J., Ayeni O., Gray R., Pinol A. (1984). Effects of Hormonal Contraceptives on Milk Volume and Infant Growth. Contraception.

[2] Taylor H.S., Pal L., Sell E., Taylor H.S., Pal L., Sell E. (2019). Hormonal Contraception. Speroff’s Clinical Gynecologic Endocrinology and Infertility.

[3] Kovacs G. (1996). Progestogen-Only Pills and Bleeding Disturbances. Hum. Reprod..

[4] Steinberg A. (2006). Menstruation. Encyclopedia of Medicine and Jewish Law.

[5] Salleh S.F., Nordin N., Ismail S.K., Muda T.F.M.T., Yusoff Z.M., Ali R.M., Alqaraleh R.S.S. (2018). Intercourse during Menstruation: Islamic Ethics and Medical Views. Int. J. Acad. Res. Bus. Soc. Sci..

[6] World Health Organization (2015). Reproductive Health and Research. Medical Eligibility Criteria for Contraceptive Use.

[7] Segev L., Samson A.O., Katz-Samson G., Srebnik N. (2023). The Risk of Breakthrough Bleeding Justifies the Use of Combined Hormonal Contraception Over Progesterone-Only Pills While Breastfeeding. Breastfeed. Med..

[8] Gosset A., Denuelle M., Valton L., Sommet A., Bénévent J., Tremollières F. (2022). Interactions between Antiseizure Medications and Contraception: A Study about the Knowledge of Patients and Their Specialist Physicians. Epilepsy Behav..

[9] Bombas T., Costa A.R., Palma F., Vicente L., Sá J.L., Nogueira A.M., Andrade S. (2012). Knowledge-Attitude-Practice Survey among Portuguese Gynaecologists Regarding Combined Hormonal Contraceptives Methods. Eur. J. Contracept. Reprod. Health Care.

[10] Lopez L.M., Grey T.W., Stuebe A.M., Chen M., Truitt S.T., Gallo M.F. (2015). Combined Hormonal versus Nonhormonal versus Progestin-Only Contraception in Lactation. Cochrane Database Syst. Rev..

[11] Espey E., Ogburn T., Leeman L., Singh R., Ostrom K., Schrader R. (2012). Effect of Progestin Compared with Combined Oral Contraceptive Pills on Lactation: A Randomized Controlled Trial. Obstet. Gynecol..

[12] Welsh A. (2015). Best Practice in Postpartum Family Planning.

[13] https://www.Acog.Org/Womens-Health/Faqs/Combined-Hormonal-Birth-Control-Pill-Patch-Ring.

[14] https://www.Nhs.Uk/Conditions/Contraception/Vaginal-Ring/.

[15] Frieden T.R., Harold Jaffe D.W., Thacker S.B., Moolenaar R.L., LaPete M.A., Martinroe J.C., Spriggs S.R., Starr T.M., Doan Q.M., King P.H. Centers for Disease Control and Prevention MMWR Morbidity and Mortality Weekly Report. 2011. Volume 60, pp. 878–883. Atlanta, GA, USA. https://www.cdc.gov/mmwr/preview/mmwrhtml/mm6026a3.htm?s_cid=mm6026a3_w.

[16] Nilsson S., Nygren K.G., Johansson E.D. (1978). Ethinyl Estradiol in Human Milk and Plasma after Oral Administration. Contraception.

[17] Betrabet S.S., Shikary Z.K., Toddywalla V.S., Patel D., Vaidya P., Saxena B.N. (1986). ICMR Task Force Study on Hormonal Contraception. Biological Activity of Ethinyl Estradiol Present in the Breast Milk. Contraception.

[18] Pinheiro E., Bogen D.L., Hoxha D., Wisner K.L. (2016). Transdermal Estradiol Treatment during Breastfeeding: Maternal and Infant Serum Concentrations. Arch. Womens Ment. Health.

[19] Lönnerdal B., Forsum E., Hambraeus L. (1980). Effect of Oral Contraceptives on Composition and Volume of Breast Milk. Am. J. Clin. Nutr..

[20] Costa T.H., Dorea J.G. (1992). Concentration of Fat, Protein, Lactose and Energy in Milk of Mothers Using Hormonal Contraceptives. Ann. Trop. Paediatr..

[21] Sausjord I.K., Acton L.W., White K.O., O’Connor S.K., Lerner N.M. (2023). Breastfeeding and Hormonal Contraception: A Scoping Review of Clinical Guidelines, Professional Association Recommendations, and the Literature. Breastfeed. Med..

[22] Tepper N.K., Phillips S.J., Kapp N., Gaffield M.E., Curtis K.M. (2016). Combined Hormonal Contraceptive Use among Breastfeeding Women: An Updated Systematic Review. Contraception.

[23] Turkyılmaz C., Onal E., Hirfanoglu I.M., Turan O., Koç E., Ergenekon E., Atalay Y. (2011). The Effect of Galactagogue Herbal Tea on Breast Milk Production and Short-Term Catch-up of Birth Weight in the First Week of Life. J. Altern. Complement. Med..

[24] Kajale N.A., Chiplonkar S.A., Khadilkar V., Khadilkar A.V. (2016). Effect of Breastfeeding Practices and Maternal Nutrition on Baby’s Weight Gain During First 6 Months. J. Obstet. Gynaecol. India.

[25] Zong X.-N., Li H., Zhang Y.-Q., Wu H.-H. (2020). Growth Performance Comparison of Exclusively Breastfed Infants with Partially Breastfed and Formula Fed Infants. PLoS ONE.

[26] Kramer M.S., Kakuma R. (2012). Optimal Duration of Exclusive Breastfeeding. Cochrane Database Syst. Rev..

[27] Sheth A., Jain U., Sharma S., Adatia A., Patankar S., Andolsek L., Pretnar-Darovec A., Belsey M.A., Hall P.E., Parker R.A. (1982). A Randomized, Double-Blind Study of Two Combined and Two Progestogen-Only Oral Contraceptives. Contraception.

[28] Vekemans M. (1997). Postpartum Contraception: The Lactational Amenorrhea Method. Eur. J. Contracept. Reprod. Health Care.

[29] Van der Wijden C., Manion C. (2015). Lactational Amenorrhoea Method for Family Planning. Cochrane Database Syst. Rev..

[30] Vilk Ayalon N., Segev L., Samson A.O., Yagel S., Cohen S.M., Green T., Hochler H. (2022). Norethisterone Reduces Vaginal Bleeding Caused by Progesterone-Only Birth Control Pills. J. Clin. Med..

[31] Danvers A.A., Gurney E.G., Panushka K., Peskin M., Evans T.A. (2023). Shortcomings and Disparities in Contraception Counseling and Use by Hypertensive Individuals at Risk for Unintended Pregnancy: A Comparative Analysis of the National Survey of Family Growth 2015–2019. Am. J. Obstet. Gynecol..

[32] Callegari L.S., Aiken A.R.A., Dehlendorf C., Cason P., Borrero S. (2017). Addressing Potential Pitfalls of Reproductive Life Planning with Patient-Centered Counseling. Am. J. Obstet. Gynecol..

